# Bilateral Vertebral Artery Dissection: A Case Report with Literature Review

**DOI:** 10.1155/2020/8180926

**Published:** 2020-05-25

**Authors:** Olga Gomez‐Rojas, Adam Hafeez, Nikhil Gandhi, Ramona Berghea, Alexandra Halalau

**Affiliations:** ^1^Office of Occupational Health, Alexander von Humboldt Peruvian German School, Lima, Peru; ^2^Division of Cardiovascular Medicine, Department of Medicine, University of Florida, Gainesville, FL, USA; ^3^Internal Medicine Department, Ascension Health, St.John Hospital, Detroit, MI, USA; ^4^Internal Medicine Department, Beaumont Hospital, Royal Oak, MI, USA; ^5^Oakland University William Beaumont School of Medicine, Rochester, MI, USA

## Abstract

Vertebral artery dissection (VAD) is a rare cause of ischemic stroke in young patients. The largely nonspecific symptoms and delayed presentation pose a serious diagnostic challenge. Medical management with either anticoagulation or antiplatelet therapy is recommended, but there are no reports of successful dual therapy. We report a case of spontaneous bilateral vertebral artery dissections (VADs) treated with both anticoagulation and antiplatelet therapy and a literature review on clinical presentation and the current medical and surgical management options. A 37-year-old healthy female presented to the emergency department with worsening neck pain and headache for two weeks despite over-the-counter medication, block therapy, yoga, and deep tissue neck massage. She denied any trauma but admitted to multiple roller coaster rides over the past few months. CT angiography was concerning for VADs, and MRI brain revealed multiple strokes in the left posterior inferior cerebellar artery (PICA) territory. Cerebral arteriography confirmed the diagnosis of VADs. The patient was initiated on warfarin, along with atorvastatin and aspirin. She was discharged home with no complications and followed up with neurology as an outpatient. MR angiography after three months revealed complete resolution of the dissection. The patient did not report any bleeding complications from dual therapy.

## 1. Introduction

Cervical artery dissection presents at any age, with a wide range of symptoms. Although uncommon in the general population, it is a recognized cause of ischemic stroke in younger patients. The internal carotid artery is most commonly affected, but occasionally, other arteries can be involved as well [1]. The incidence of vertebral artery dissection is estimated to be 2.6–3/100 000 [2]. According to Schievink, bilateral VAD accounts for 10 to 25% of all causes of ischemic stroke in young patients [3]. Bilateral VAD may be fatal due to the potential compromise of the posterior cerebral circulation.

Early diagnosis is crucial to achieve better outcomes, especially when the presentation is vague or asymptomatic. Headaches, neck pain, and dizziness are common complaints seen in young to middle age individuals presenting to outpatient clinics and emergency centers. These symptoms can be attributed to benign conditions such as migraine, tension headaches, and myofascial or musculoskeletal pain; therefore, the diagnosis of VAD is frequently missed or misdiagnosed [4]. A common theme amongst VAD is patients with recent minor neck trauma or maneuvers damaging the underlying cervical vessels, including chiropractic manipulation [5].

The VAD diagnosis is based on a high index of suspicion combined with various imaging modalities. Therefore, this case would be of high interest to both general practitioners and emergency medicine physicians, who most frequently deal with unspecific head and neck pain. Management of VAD depends on several factors including clinical presentation, time of onset, anatomic findings, number of vessels compromised, and contraindications to specific therapies. Therapeutic options include thrombolysis, conservative management with antithrombotic drugs (antiplatelet or anticoagulation agents), endovascular management, and surgery. Timely diagnosis and initiation of treatment can help prevent more serious complications. Prognosis appears promising, as recurrent dissection rate after the first month is approximately 1% per year [6]. We report the case of a young female who presented 2 weeks after initiation of neck pain. The diagnosis of VAD and multiple strokes was made. We highlight our management approach and outcome. Additionally, we present a review of literature on the most common clinical presentations and management options available to date.

## 2. Case Presentation

A 37-year-old Caucasian female with a history of dyslipidemia, asthma, and fibroids presented to the emergency room reporting two weeks of bilateral posterior neck pain and headaches. She initially presented with right-sided neck pain, which she attributed to various physical activities and sleeping in uncomfortable hotel beds. Of note, she described multiple recent visits to a theme park and enjoyed numerous roller coaster rides. The pain was achy, constant in nature, extending to the back of the eyes, and rated as 4 on a 10-point scale. Her pain persisted and increased in severity despite acetaminophen use and application of heating pads. She then tried block therapy, yoga, and deep tissue neck massage. The pain then became bilateral, even more severe, followed by an episode of severe dizziness and nausea which prompted the emergency room visit. Her family history was negative for connective tissue diseases. The patient was taking oral contraceptive pills and had discontinued statin therapy a few years ago. She denied using tobacco products, illicit drugs, or excessive alcohol.

Initially, vital signs were within normal limits. Physical exam revealed a nontoxic appearing young woman in no visible distress. Neck exam was significant for muscular tenderness but no midline or cervical spinous process tenderness. There was no rigidity, with full active and passive range of motion without pain. Brudzinski and Kernig signs were negative. The ear canals showed no abnormalities; there were no hearing deficits. No carotid bruits were present, and the neck was supple. Neurological exam revealed a normal cranial nerve exam, +5/5 muscle strength in the upper and lower extremities symmetric bilaterally, normal sensory exam, normal finger-to-nose and heel-to-shin testing, and no dysdiadochokinesia. Gait was intact, and she had no dysarthria or nystagmus.

Based on her history of recent roller coaster rides, worsening neck pain after yoga, and deep tissue neck massage, the following differential diagnosis was considered: intracranial bleeding, stroke, cervical artery dissection (CAD), and cervical spondylosis. Complete blood count and urinalysis were unremarkable. Basic metabolic panel showed abnormal cholesterol levels (total cholesterol: 224 mg/dl, triglycerides: 168 mg/dl, and LDL: 131 mg/dl). Beta-HCG was <2, and troponins were negative. ECG showed normal sinus rhythm. Autoimmune and vasculitis workup showed an elevated sedimentation rate of 27 mm/hr [0–22 mm/hr] but negative antinuclear antibodies, Smith antibody, and RNA antibody. Rheumatoid factor and C-reactive protein were within normal limits.

**Figure 1:**
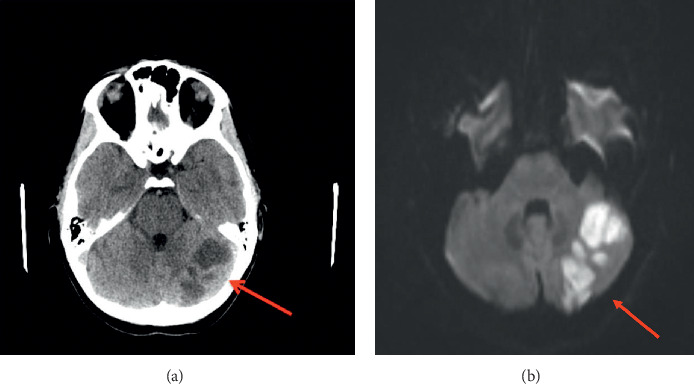
Noncontrast CT of the head (a) and brain MRI-DWI sequence (b) showing several well-circumscribed near fluid attenuation regions in the left cerebellum, the largest of which measures 3.5 cm transverse diameter. This is suggestive of left cerebellar ischemic stroke. There is no acute hemorrhage, midline shift, or hydrocephalus.

**Figure 2:**
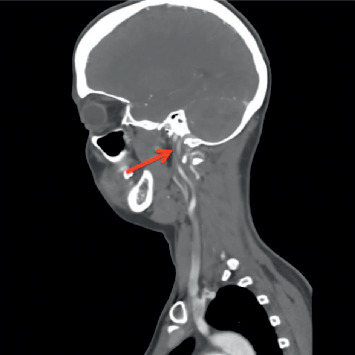
CTA shows a short segment of high-grade narrowing of the cervical right vertebral artery at the C2-3 level.

**Figure 3:**
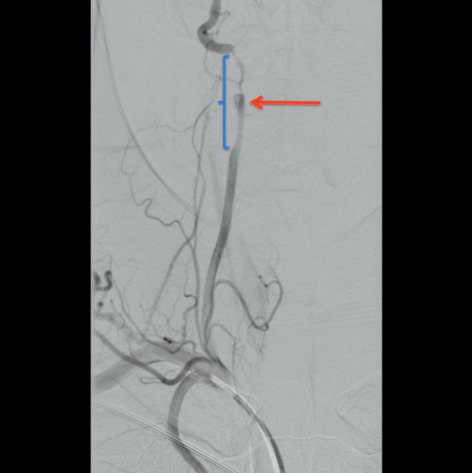
Cerebral angiography showing a dissection flap extending across the C1 loop and to the foramen magnum. Subintimal clot is seen medially in the vessel wall with 50% loss of lumen in the V4 segment. This abrupt change in the caliber of the left vertebral artery shows dissection.

**Figure 4:**
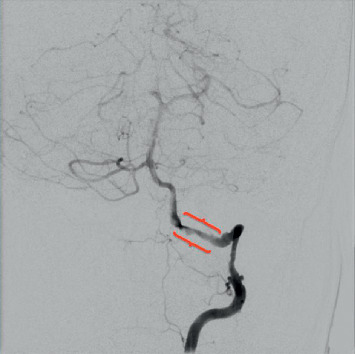
Cerebral angiography shows right vertebral artery dissection right past the C3 level at which a small pseudoaneurysm is noted in the artery anterior wall. This pseudoaneurysm tapers into the dissection which extends just beyond the C2 loop. There is marked reduction in luminal diameter and noticeable delay in antegrade flow distal to the dissection.

Chest X-ray was unremarkable. CT head/brain without IV contrast showed nonspecific left cerebellar hypodensities that may represent age-indeterminate embolic infarcts/injuries among other possible etiologies, with no acute intracranial hemorrhage (Figures 1(a) and 1(b)). Head MRI without contrast (DWI sequence) showed large areas of acute ischemia in the left posterior inferior cerebellar artery (PICA) territory with additional smaller areas of acute ischemia in the left superior parasagittal cerebellum. CT angiography of the head and neck with and without IV contrast demonstrated a relatively abrupt change in the caliber of the left V3 vertebral artery suggestive of dissection and a short segment high-grade narrowing of the right vertebral artery also highly suggestive of a dissection (Figure 2). Carotid arteries were within normal limits. Twenty-four hours after admission, the patient presented with a second episode of dizziness, vertigo, nausea, vomiting, and tingling sensation in both hands, which prompted further evaluation with the cerebral angiogram. The exam confirmed the bilateral VAD and revealed the presence of a subintimal clot on the left VA at V4 with loss of 50% of lumen (Figures 3 and 4), not previously observed in previous images. MR angiogram was not performed in the initial evaluation.

The patient was evaluated conjointly with neurology, neurosurgery, and interventional neuroradiology. Upon diagnosis of bilateral VADs, all consultants unanimously recommended systemic anticoagulation for 3–6 months and repeat imaging in 3 months to evaluate for healing. The patient was not a candidate for thrombolytic therapy given the time of presentation and NIHSS of 0. Intravenous heparin at 1,000 units/hr without initial bolus and close monitoring was preferred over standard therapy due to the high risk of hemorrhagic transformation. Additionally, aspirin and atorvastatin were also started. The patient developed mild vaginal bleeding upon discontinuation of birth control pills. The episode stopped shortly after and no interventions were needed. Mirena intrauterine device was recommended should the bleeding worsen while on anticoagulation but was never placed as the bleeding stopped shortly after.

**Figure 5:**
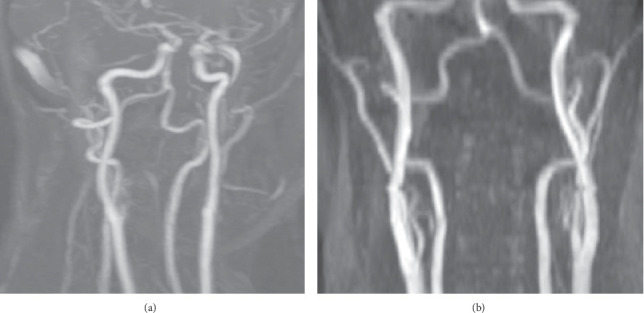
MR angiogram at 3-month follow-up showed complete resolution of bilateral dissection.

A second neurology opinion, obtained per patient request, recommended to continue systemic anticoagulation for 3–6 months along with aspirin and atorvastatin. The patient was continued on IV heparin and titrated carefully for activated partial thromboplastin time (aPTT) goal of 50–55 seconds. She was given aspirin 325 mg daily initially and then decreased to aspirin 81 mg when aPTT became therapeutic. Atorvastatin 10 mg was continued throughout the hospital stay, without complication. The patient was eventually bridged to warfarin with an international normalized ratio (INR) goal of 2–3 with intention to continue for 3–6 months. At discharge, the patient continued with both low dose aspirin and warfarin without complications or neurological deficits. She follows up with neurology as outpatient. INR was monitored biweekly and remained within therapeutic range throughout the 3 months. MR angiography after three months showed complete resolution of the dissection (Figure 5). The patient did not report any bleeding complications from dual therapy.

## 3. Literature Review

A systematic review through PubMed was conducted without any restrictions. We used the keywords: “bilateral,” “vertebral artery,” and “dissection.” The reference sections of the eligible results were screened for any potential missed cases of relevance to ensure the inclusion of all reported cases of bilateral vertebral artery dissection. Our search yielded one hundred and one manuscripts. Exclusion criteria included artery transections, unilateral dissections, dissection secondary to known aneurysms, congenital or acquired vasculopathies, dissection in arteries with anomalous origin, patients with connective tissue disorders, cases presenting with subarachnoid hemorrhage, pediatric patients, patients with chromosopathies, articles other than case reports or case series, and articles not available in English. Thirty-two manuscripts describing cases of bilateral vertebral artery dissection in patients were included in our review and are summarized in Table 1.

In our review, the age of presentation ranged between 23 and 52 years old. Of the thirty-two cases, eighteen (56.2%) were female patients. These baseline characteristics were consistent with previous reports [3, 14]. Among the identifiable risk factors for vertebral dissection, we found five patients presenting with associated comorbidities: one patient with chronic hypertension, three with a history of migraines, and one with acute demyelinating encephalomyelitis [14, 17]. The remaining cases were otherwise healthy young adults. Thirteen cases of bilateral VA dissection (40.6%) presented spontaneously and without an identifiable trigger. Among those with a potential source of trauma, 6 patients were associated with chiropractic manipulation (Cases 1a, 4, 13, 17, 21, and 28), 6 to motor vehicle accident (Cases 3, 5, 6a, 11, and 12), 4 to sports with or without direct trauma (Cases 10, 14, 22, and 26), and one case followed 2 weeks postpartum (Case 25). Similar to our case, there were 2 cases associated with recent use of roller coasters (Cases 16 and 19).

The most common clinical presentations were occipital headache, unilateral or bilateral neck pain, and vertebrobasilar symptoms (dizziness, nausea, and vertigo). Two patients with multivessel compromise that included bilateral internal carotid artery dissection also presented with monocular vision loss or gaze deviation (Cases 19 and 25). The least common presentations were three cases of locked-in syndrome, one case of Horner syndrome, and one case of Brown-Sequard syndrome (Cases 3, 5, 6, and 9). In these cases, there was an extended compromise of the basilar artery, bilateral internal carotid arteries, or thoracic vertebral artery. Of all reported cases, one patient died 2 weeks after the development of symptoms (Case 1b).

Anticoagulation therapy with heparin was started in all nonsurgical patients once any contraindications were ruled out. One patient with extensive compromise of the posterior circulation required immediate thrombolytic therapy (Case 22). In this case, clinical deterioration followed, and the patient underwent suction thrombectomy 24 hours later. Follow-up 12 months after surgery showed clinical improvement with residual right incomplete hemianopsia. In another patient, stent placement was indicated given multiple vessel involvement, severe compromise of posterior circulation, and high risk of distal embolization (Case 25).

At discharge, most patients continued anticoagulation with warfarin for at least 3 months. The addition of other drugs and duration of treatment were variable. Chakrapi et al. reported use of prolonged anticoagulation with warfarin for six months, clopidogrel for eighteen months, and aspirin indefinitely. For most cases, follow-ups 3 to 6 months after the initial episode showed clinical improvement and partial to complete resolution of the arterial dissections.

## 4. Discussion

We report a case of bilateral VAD with embolic strokes in a young woman presenting with worsening posterior neck pain, headaches, and vertigo. We suspect that exposure to constant repetitive microtrauma during roller coaster rides may have caused the dissections, with subsequent deep tissue massage and block yoga mobilizing intramural thrombus causing the subclinical strokes.

Neck pain remains a very common complaint among patients. Approximately 10% of adults experience neck pain at any time, but only about 1% will develop further neurologic symptoms [37]. Common causes of neck pain include cervical strain, internal disc disruption, cervical facet-mediated pain, “whiplash” syndrome, and myofascial pain [38]. Clinical symptoms that are persistent, worsening, or are associated with neurological deficits may suggest more serious causes.

An arterial dissection can occur at any age, and it is a recognized cause of stroke, especially in younger patients [39]. The false lumen produced by the dissection can cause luminal stenosis or thrombosis, compromising the blood flow. A retrospective study of patients with cervical artery dissections found that, in patients with acute stroke, 85% was attributed to the thromboembolic mechanism, while only 12% was attributed to hypoperfusion [40]. In young adults, the cases of nontraumatic bilateral cervicocephalic artery dissections are less common but not rare. About 5–10% of carotid and 38% of VAD are bilateral. In a case series, bilateral spontaneous dissection was found in 8.1% of all patients with VAD [41].

In a review of 83 patients with unilateral vertebral artery dissection, without consciousness disturbance at admission, unilateral or bilateral headache was the most common complaint (60 cases), followed by neck pain (41 cases), and vertigo (20 cases). Statistically, unilateral headache and/or neck pain was more common in cases with definite vertebral artery dissection compared with other classifications per the Spontaneous Cervicocephalic Arterial Dissections Study (*P* = 0.040) [42]. In the thirty-two cases of bilateral VAD we reviewed, 62.5% (20 patients) presented with either head or neck pain as the chief complaint. Ten cases reported involvement of other vessels: bilateral internal carotid in Cases 13, 17, 19, 21, 23, and 15; basilar artery in Cases 15 and 18; and multiple vessels in Cases 9 and 22. Therefore, medullary and pontine symptoms such as vertigo, dysphagia, and Horner syndrome predominated in these cases.

An analysis of a national trauma database showed that VADs only comprised 0.01% of all patients admitted with head and neck trauma [43]. Observational studies have produced a list of mild mechanical triggering events. Relevant to our case, amusement park rides have been implicated in VADs. Schneck et al. reported a case of a 34-year-old female who was misdiagnosed with labyrinthitis after presenting to her primary care physician complaining of vertigo. She had recently been to a national amusement park where she went on all but one ride. Similar to our case, the patient developed neck pain uncontrolled with analgesics and subsequently developed neurologic symptoms of blurry vision. Magnetic resonance angiography revealed bilateral VAD without infarction [22]. Our patient's dissection was complicated by multiple strokes, most notably in the left PICA territory but fortunately without any evidence of Wallenberg syndrome, Horner syndrome, or gait disturbances.

Multiple dissections are more frequently found in women [44]. Arnold et al. used prospective hospital-based registries to identify risk factors and outcomes in patients with spontaneous cervical artery dissections. They found that 1.5% of patients had multiple dissections, and the majority was women. Additionally, these patients did not have any family history of such dissection, fibromuscular dysplasia, or connective tissue disease [45]. This is similar to our patient who had two dissections on imaging and no identifiable risk factors.

Traditionally, VADs are classified as extracranial and intracranial. Extracranial dissections in the hyperacute period can be treated with thrombolytic therapy. Outside said period, antithrombotic therapy with either single or dual antiplatelet or anticoagulation agents is the treatment of choice. VAD management remains challenging as there are no conclusive treatment guidelines. Anticoagulation therapy is controversial in intracranial dissections given the theoretical higher risk of bleeding [46]. Per the Journal of the American College of Cardiology, antithrombotic treatment with an anticoagulant or platelet inhibitor for at least 3–6 months is reasonable for patients with extracranial carotid or vertebral artery dissection associated with ischemic stroke or TIA (level of evidence: B) [47]. However, there is no clear consensus regarding management for the use of anticoagulation over antiplatelet therapy, or both, in patients without atherosclerotic disease in vertebral artery dissection.

Most recently, the CADISS trial compared antiplatelet treatment with anticoagulant treatment for extracranial carotid and vertebral artery dissection. It showed that recurrent stroke at 3 months is rare, with no significant difference between the two treatment groups. Although more strokes occurred in the antiplatelet group, this difference was counterbalanced by one major subarachnoid hemorrhage in the anticoagulant group [48]. Based on these results and several retrospective studies, the American Heart Association/American Stroke Association recommends either antiplatelet or anticoagulation therapy for 3 to 6 months in patients with CAD-associated ischemic stroke (class of recommendation IIa, level of evidence B-NR) [49]. Some authors may favor antiplatelet treatment given its safer profile and the risk of warfarin inducing expansion of the intramural hematoma [50]. However, this and other complications such as hemorrhagic transformation are rare events. Our literature review indicates that most physicians gravitate towards early anticoagulation with heparin and then transitioning to oral warfarin. This action may be supported by the theoretical benefit of anticoagulation in the prevention of occlusion of a stenotic vessel and minimization of distal embolization [46, 51, 52].

The combination of antiplatelet and anticoagulation therapy is controversial, as it increases the hemorrhagic risk. However, even in the setting of hemorrhagic transformation, antithrombotic therapy could be continued based on the individual risk [49]. In this patient, we took into consideration her past medical history (dyslipidemia and recent OCP use), low risk of bleeding (Has-bled score of 2), the high grade, bilateral compromise of the vertebral arteries, presence of a subintimal clot in close proximity to the basilar artery, and acute embolic strokes. Our approach allowed for management of her risk factors, prevention of recurrent strokes, and potential lethal compromise of the basilar artery by distal embolization. In this particular case, the authors concluded that the benefits of such therapy outweighed the risks. To our knowledge, this is the first report of dual antiplatelet and anticoagulation therapy in bilateral VAD. The patient was closely monitored in a neurologic progressive unit with serial neurovascular assessment without any eventualities. Upon discharge, she continued with the same regiment, and no adverse events were reported.

There are no concrete data on the ideal duration of antithrombotic therapy. Current recommendation is to continue antithrombotic therapy for 3 to 6 months, as the vessel heals. Further treatment should be tailored based on imaging findings [46]. Chakrapi et al. reported one case of bilateral ICA and VA dissection that was managed with prolonged anticoagulation therapy plus dual antiplatelet therapy. At 6 months follow-up, the patient was asymptomatic, and warfarin was discontinued. The 1-year CT scan results showed residual dissection of 30% in the right ICA and persistent 30 to 50% dissection in both VA. For these reasons, the patient continued clopidogrel for 12 months (18 months in total since the episode) and aspirin indefinitely [23].

Some studies are proposing the use of new oral anticoagulant agents (NOAC) as an attractive therapeutic alternative. A recent retrospective observational study compared the use of NOAC to conventional anticoagulation and antiplatelet therapy in patients with CAD. The results show that NOAC have similar rate of recurrence of stroke, lower risk of major bleeding, but higher risk for radiologic worsening than conventional anticoagulation and antiplatelet therapies. Additionally, two case reports showed complete resolution of the lesion at 3 and 6 months follow-up [53, 54]. In one of the reports, there were two cases of minor bleeding (hemorrhoidal and metrorrhagia), none of which required discontinuation of therapy.

NOAC have been found safer than warfarin in preventing stroke in patients with nonvalvular atrial fibrillation [55]. In addition to their safety profile, NOAC may be a more attractive choice for younger patients who are socially active, as there is no need for continuous monitoring of medication or interference with diet and lifestyle. On the contrary, titration is required in older patients with renal dysfunction [56]. However, randomized clinical trials should be conducted to determine if NOAC are effective and safe in this population.

Endovascular management (EM) is considered in patients with progression of disease despite antithrombotic treatment, those presenting with pseudoaneurysm or in those not amenable for anticoagulation [50]. The safety and efficacy of EM have been explored in several retrospective studies, with overall good outcome profiles [57, 58]. In a retrospective study, 140 patients with CAD were treated with stent placement. In the follow-up period, imaging showed improvement of the vascular stenosis. Additionally, only 1.4% of the patients presented with stroke events after the intervention [57]. A meta-analysis of 39 retrospective studies involving different treatment modalities for VADs in adults was done to determine clinical outcomes of patients who were treated endovascularly. Overall, 75.11% had excellent outcomes, 10.10% had good outcomes, and 13.70% had poor outcomes. Of significance, postoperative complications occurred in 10.52%, with 2.73% exhibiting vasospasm, 3.03% experiencing postoperative rebleeding, and 6.31% developing ischemia [48]. While most studies showed clinical and radiologic improvement of the vascular lesion and an acceptable rate of complications [59], conservative medical management remains the treatment of choice. Current indications for EM in CAD include recurrent ischemic events, high-grade arterial stenosis dissection with significantly limited flow, expanding pseudoaneurysm associated with dissection and occlusion, and failed medical treatment [58]. Surgical treatment may be indicated when the patient presents with recurrent strokes despite medical treatment and failed or not a candidate for endovascular therapy [49].

In our review, two patients required surgical intervention [31, 60]. The first patient presented with worsening symptoms 24 hr after thrombolytic therapy, requiring suction thrombectomy [60]. The second patient had dissection of multiple cervical vessels and severe compromise of the posterior circulation. For these reasons, three overlapping stents were placed in each vertebral artery. Both patients recovered successfully with minimal sequelae. When choosing a treatment modality, whether it is operative versus nonoperative, complications for each should be taken into careful consideration.

Per AHA guidelines, the management of additional risk factors should take place as soon as possible. Dyslipidemia is a well-known cardiovascular risk factor, and its management with statins has become a staple in ischemic strokes secondary to atherosclerotic disease. The role of dyslipidemia in the pathophysiology of artery dissection is less clear. Gupta et al. reported that traumatic dissections of the cervical arteries are more likely to occur in a vessel exposed to vascular risk [61], and Yamada et al. reported correlation between dyslipidemia and vascular repair [62]. However, most patients with VAD are young and present without classic cardiovascular risk factors. We considered statin therapy appropriate in this patient given her particular risk factors (history of dyslipidemia and traumatic VAD).

There are not any evidence-based recommendations on the restrictions or continuation of physical activity after an episode of CAD. Although participation in sports has been reported in many cases, causation has not been demonstrated. The recurrence of CAD is uncommon, with a reported incidence of 2% within the first month of the event and a year incidence of 1% after that [63]. This information is insufficient to draw any conclusions regarding the role of physical activity in the recurrence of CAD. Some experts recommend patients with dissection to avoid contact sports, chiropractic manipulation, and activities that involve abrupt mobilization of the neck [46]. Prospective studies to determine the effect of different levels of physical activity on the recurrence of CAD may be unfeasible, given the low rate of recurrence. In this case, the patient was advised to avoid any contact sports, manipulation of the neck, yoga, and roller coasters.

## 5. Conclusion

This case was written to raise awareness for health care providers, especially general practitioners and emergency department physicians, regarding injuries in the adult population after amusement park rides and/or other physical activities which involve excessive, forceful, or repetitive movements of the neck. We report a case of bilateral VAD complicated by embolic strokes in a young patient presenting with worsening posterior neck pain and headaches. These general complaints are unspecific and common in previously healthy patients. This diagnostic challenge requires an astute clinician to combine symptoms with key historical elements. Since patients can present immediately after the event or several days later when conservative management has been unsuccessful, it is imperative to keep a broad differential diagnosis in patients with neck pain that is nonresponsive to initial conservative management. We insist on the importance of high clinical suspicion to facilitate an early diagnosis.

Management-wise, most cases treated with anticoagulation therapy resolve in the following six to twelve months period without major neurologic deficits, especially in absence of intracranial vessel compromise. There is no consensus on whether antiplatelet therapy is superior to anticoagulation therapy, but current evidence favors the use of antiplatelets in the cases of intracranial dissection and extracranial dissection without ischemic symptoms. If there is evidence of thrombus or severe vessel occlusion, anticoagulation therapy may be preferred in order to prevent distal embolization. Our review showed that most physicians opt for anticoagulation with heparin during the intrahospital stay and bridge to warfarin at discharge. Antithrombotic therapy duration has not been defined yet, but it is usually carried for 3 to 6 months or until evidence of a well-established healing or resolution of the dissection. Reports on the use of NOAC show initial positive outcomes, but larger prospective studies are required before reaching definitive conclusions. The addition of other drugs such as atorvastatin, aspirin, or clopidogrel is left to the physician's discretion and the patient's individual risk factors. Successful management with endovascular and surgical procedures has been reported in several cases, but their safety and efficacy are yet to be proven in randomized clinical trials. There are not any evidence-based recommendations on the restrictions or continuation of physical activity after an episode of CAD. Experts' advice is to avoid contact sports, high-risk activities, and neck manipulation.

## Figures and Tables

**Table 1:** Reported cases of bilateral vertebral artery dissection.

Case #	Age/sex	Risk factor	Trigger	Clinical presentation	Vessel	Imaging	Medical management	Surgical management	Outcome	Comments
(1) Katirji et.al. [7]	(1) 26 F (2) 34 M	(a) None (b) None	(a) Fall plus chiropractic manipulation; (b) none	(1) Neck pain, headache, vertigo, nausea, and vomiting (2) Occipital headache, subsequent locked-in syndrome	(1) Angio: bilateral VA dissection at C1–C6 (2) Autopsy: RVA and LVA thrombosis	(1) CT: right cerebellar aneurysm. (2) CT: bilateral cerebellar and pontine infarct	(1) Heparin, dexamethasone, human plasma protein fraction, and warfarin (2) None	None	(1) Residual right-sided ataxia (2) Patient died after 2 weeks	Collected from a 5-case report
(2) Leys et al. [8]	35 M	None	None	Vertigo, diplopia, right limb weakness, and occipital pain	Angio: RVA with double lumen and irregular stenosis of LVA	CT scan: unremarkable	Heparin for 2 months and then aspirin 500 mg	None	At 1 year: persistent left miosis and right hypoesthesia to temperature and pain	—
(3) Rae-Grant et al. [9]	31 M	None	MVA	Occipital headache, nausea, and progression to locked-in syndrome	Angio: RVA occlusion at C1, C2; LVA unperfused beyond PICA	MR: pontine SAH, right cerebellar infarct	Supportive care	None	Extraocular impairment and paresis of four extremities	—
(4) Philips et al. [10]	39 M	None	10 days course of chiropractic manipulation for neck pain	Dizziness, speech disturbance, and left-sided weakness than progressed to pure motor deficit of the left side	Angio: LVA occlusion at C6. RVA irregular stenosis through foramen transversarium	CT: right-sided midpontine infarct	None; patient transferred to final facility 4 days after stroke when symptoms were improving	None	Partial motor improvement; dependent on cane and drop-foot splint	—
(5) Fox and Lavin. [11]	30 M	None	MVA	Occipital headache, vertigo, and progression to locked-in syndrome	Angio: absence of flow above C1in both vertebral arteries	MR: pontine infarct, right cerebellar infarct with SAH	Heparin	None	Speech impairment and ambulation with assistance	—
(6) Hinse et al. [12]	(1) 39 F (2) 28 F	1. None 2. Chronic hypertension	(a) MVA (b) None	(1) Right neck pain, vertigo, dysarthria, right Horner, and left hemiparesis (2) Vertigo, unsteady gait	(1) DSA: RVA stenosis at C1-C2, irregularities in LVA (2) DSA: RVA stenosis at C1-C2; LVA occlusion at C1	(2) CT: left PICA/cerebellar infarct	(1) Heparin and phenprocoumon (2) Heparin and coumarin	None	(1) Hemisensory deficit (2) No neurologic deficits	Collected from a 4-case report
(7) el Nakadi. et al. [13]	35 F	DM	None	Several transient episodes of headache, nausea, ataxia, and sensory loss	DSA: RVA stenosis with double lumen. LVA occlusion at C1	MR: left occipital and right cerebellar lobe infarcts	Antiaggregation therapy: not specified	None	Fluctuant ischemic symptoms with persistent occlusion of RVA after 1 year.	—
(8) Chang et al. [14]	29 M	None	None	Headache, left facial and perioral numbness, tingling of the right upper extremity, vertigo, ataxia, hoarseness, and dysphagia	Angio: RVA stenosis at C1-C2, LVS stenosis at the foramen magnum	MR: PICA infarct	Heparin, warfarin	None	Speech impairment	—
(9) Garnier et al. [15]	45 F	None	None	Atypical, right cervical Brown-Sequard's syndrome	Angio: bilateral vertebral artery dissection	Angio: irregular stenosis of the right and left cervical vertebral artery	Anticoagulation: not specified	None	1 yr follow-up: spastic paraparesis with right-sided central pain and mild urinary retention; MRI and MRA showed the resolution	No specification of anticoagulation drug of choice
(10) Karnik et al. [16]	49 F	None	Inline skating without microtrauma	Left side neck pain	MR angio: LVA dissection. MR angio 4 weeks later: RVA dissection	MR 4 weeks after: bilateral medullary infarcts	Heparin	None	At 6 months follow-up: no neurologic deficits	—
(11) Medhkour and Chan [17]	52 F	None	MVA	GCS score 14 (confused)	Angio: RVA pseudoaneurysm and occlusion at C5. LVA occlusion at C5	CT and MR: unremarkable	Heparin	None	Currently without neurologic deficits	—
(12) Taylor. and Senkowski. [18]	46 M	None	MVA	GSC score 3	Angio: bilateral VA disruption with no posterior collateral circulation	CT: basilar cistern edema	Supportive care	None	Patient died after withdrawal of ventilation	—
(13) Nagdir et al. [19]	34 M	None	Chiropractor neck manipulation	Left side loss of coordination and involuntary movements, dysarthria, and hypoesthesia	MR angio: bilateral internal carotid dissection, LVA dissection, and RVA pseudoaneurysm at C1/C2	MR: multiple thalamic infarcts	Heparin	None	Partial resolution of symptoms with left-sided hemianesthesia	—
(14) Nagurney et al. [20]	23 F	Migraines	Strenuous exercise sessions	Several episodes of self-resolving occipital pain associated with visual disturbances: blurry vision, scintillations, and transient visual field loss	CT angio: bilateral vertebral artery dissection	MR brain: unremarkable	Heparin for 10 days; oral anticoagulation with warfarin for 6 months	None	Follow-up at 6 months: images showed complete resolution	—
(15) Hagiwara et al. [21]	48 M	None	Sleeping in a bad position	Severe occipital headache for 7 days and high blood pressure	MR angio: disappearance of signal flow with filling defects in the bilateral VA and BA	CT and MR unremarkable for ischemic or hemorrhagic changes	Anticoagulation: heparin bridge to warfarin	None	Not specified	—
(16) Schneck et al. [22]	33 F	None	Roller coaster	Initial head and neck pain progressed to blurry vision and right monocular vision loss in 14 days	Angio: bilateral dissection of vertebral arteries	MR unremarkable for ischemic or hemorrhagic changes	Anticoagulation: Heparin bridge to warfarin for 6 months.	None	6 months follow-up: patient was asymptomatic	Patient was changed to aspirin 325 mg after 6 months
(17) Chakrapani et al. [23]	50 F	None	Face and neck massage	Initial head and neck pain; 13 d: sudden left side neck and retro-orbital pain; ptosis and miosis	MR angio: RVA narrowed from C3 to C5, LVA from C2 to C5, and bilateral ICAs hematomas	MR was unremarkable for ischemic or hemorrhagic changes in the brain	Anticoagulation: heparin bridge to warfarin plus clopidogrel plus aspirin	None	6 months follow-up: patient was asymptomatic; 1 yr CT scan: right ICA residual dissection of 30%; left ICA dissection resolved; persistent 30 to 50% dissection of VA bilaterally	Patient stopped warfarin at 6 months; continued with clopidogrel for 18 months and aspirin indefinitely
(18) Preul et al. [24]	33 F	None	Chiropractor maneuver	Acute onset headache after snapping sensation; generalized seizures; later on: nystagmus to the right and right-sided hemiataxia and Babinski's sign	MR angio: bilateral; VA dissection with progression to the BA	MR revealed acute infarctions in the cerebellum and pons on the right and in the left thalamus and the posterior limb of the internal capsule	Anticoagulation with heparin bridge to warfarin	None	3mo: walk independently; 3 yrs: symptom free	6mo MRI: residual aneurysm of the left extracranial vertebral artery
(19) Ozkan arat et al. [25]	35 F	None	Roller coaster ride	Right side neck pain, transient right monocular vision loss	Angio: bilateral internal carotid and vertebral artery dissection	Brain imaging studies not reported	Anticoagulation therapy: heparin bridge to warfarin for 3 months	None	At 3 months: stable; no strokes, no further neurologic deficit	—
(20) Shibata et al. [26]	51 M	None	None	Sudden onset of occipital pain; progressive quadriplegia more pronounced in lower limbs and bilateral hearing loss and vertigo	CT angio: bilateral vertebral artery dissection	MR brain: unremarkable	Anticoagulation with heparin followed by oral antiplatelet therapy (aspirin)	None	1 month follow-up: remaining gait disturbance	—
(21) Koleilat et al. [27]	23 F	None	MVA	Left hemiparesis, dysarthria	CT angio: bilateral internal carotid and vertebral artery dissection	CT showed right caudate head and basal ganglia infarction	Anticoagulation with heparin and warfarin at discharge	None	Ambulates independently, residual left-sided weakness; maintained on aspirin 81 mg daily after completing 1 yr of warfarin therapy	Repeat CT of the head and neck at 32 months reveals persistent but nonocclusive dissection of bilateral carotid arteries
(22) Frankowska et al. [28]	33 M	None	Squash game without direct trauma	Headache, nausea, vomit, and right homonymous hemianopsia	CT angio: bilateral vertebral and medial occipital artery occlusion; emergency angio after rt-PA: thrombi in the basilar artery and LVA	Perfusion CT: ischemic areas in the left occipital lobe	Rt-PA; heparin after CT control 24 hr; switch to warfarin for 6 months	Suction thrombectomy	At 12 months follow-up: patient stable with right marginal incomplete hemianopsia	—
(23) Richard et al. [29]	30 M	Migraines	None	4 months of headache resistant to treatment; furtive vertigo and 1 month of permanent paralysis of the right arm	US and CT angio: bilateral vertebral and bilateral ICAs dissection	MR brain: small bilateral cerebellar infarct; MR spine: right posterior cord cervical infarct	Anticoagulation with heparin, bridged to warfarin for 3 months	None	Follow-up at 3 months: partial resolution of symptoms; MR angio control: persitent LVA occlusion; RVA and ICAs stable	Anticoagulation therapy was replaced by aspirin 160 mg for 9 months
(24) Villella et al. [30]	37 F	None	None	Sudden onset and persistent headache, nausea, and vertigo for 2 weeks despite pain medication	CT angio: bilateral vertebral artery dissection.	CT and MR: unremarkable	Anticoagulation, not specified	None	Not specified	—
(25) Goyal [31]	28 F	None	2 weeks postpartum	Right hemiparesis, gaze deviation, and mixed aphasia	DSA; bilateral internal carotid and vertebral artery dissection	CT: showed ischemic changes in the right parieto-occipital lobe	Bolus dose of heparin intravenously followed by a loading dose of clopidogrel (Plavix)	Three overlapping stents were deployed in the left ICA and 3 more in the right ICA	Poststenting angiography demonstrated successful reconstruction of the cervical ICAs; CT angiography at 6 months demonstrated complete patency of the stents and healing of the vertebral dissections	—
(26) Mas et al. [32]	30 M	None	Basketball game	Severe dysphagia, right hemiparesis, and balance dysfunction	MR angio: bilateral vertebral artery dissection	MR: multiple cerebellar infarcts	Anticoagulation, not specified	Gastrostomy 2/2 severe dysphagia	Data not available	—
(27) Stirn et al. [33]	28 F	Acute demyelinating encephalomyelitis	ADEM flare (proposed by the authors)	2 weeks of bilateral nuchal pain, left side motor deficit (hemiparesis)	MR angio: bilateral intramural hematoma of the vertebral arteries	1st MR brain (2 weeks before episode): ischemic lesion in the right parietal lobe without signs of vessel damage; 2nd MR brain: increased lesion in the same area	Anticoagulation not specified; 2 cycles of corticosteroids IV (5 days of 2gr)	None	Follow-up 11 months: resolution of symptoms, slow remission of the intracranial lesion	Autoimmune inflammation of vessels proposed as trigger for spontaneous bilateral VA dissection
(28) Ke et al. [34]	36 M	None	Chiropractic manipulation	Neck pain, right side hemiplegia and hemiparesis, progression to locked-in syndrome	CTA: bilateral vertebral artery dissection	MR brain 17 d after the event: pontine ischemia	Antiplatelet therapy after embolectomy (drug of choice not specified)	Embolectomy	Discharge at day 27: partial resolution of symptoms; persistent left side weakness and hyper-reflexia	—
(29) Lovencric [35]	40 F	Migraines, low BMI, and recent infection	None	Generalized weakness, neck pain, dizziness, and headache for 10 days plus transient visual disturbances	MR angio: bilateral vertebral artery dissection	CT and MR: bilateral ischemic lesions in the posterior circulation	Anticoagulation with LMWH and bridge to warfarin	None	Symptoms reverted 2 weeks after starting anticoagulation; no information on further follow-up	—
(30) Peters and Engelter [36]	35 F	None	None	Neck pain, ataxia, and imbalance	MR angio: bilateral distal vertebral artery dissection	MR: bilateral cerebellar infarcts, larger on the right side	Warfarin for 12 months	None	At 12 months: asymptomatic	—

Angio: angiogram; VA: vertebral artery; RVA: right vertebral artery; LVA: left vertebral artery; CT: computed tomography; MVA: motor vehicle accident; PICA: posterior inferior cerebellar artery; MR: magnetic resonance; SAH: subarachnoid hemorrhage; DSA: digital subtraction angiography; MR angio: magnetic resonance angiography; GCS: Glasgow coma scale; BA: basilar artery; ICAs: internal carotid arteries; rt-PA: recombinant tissue plasminogen activator; US-Angio: ultrasound; LMWH: low molecular weight heparin.
